# Supplementary material for: Structure of the human systemic RNAi defective transmembrane protein 1 (hSIDT1) reveals the conformational flexibility of its lipid binding domain
Source: bioRxiv. 2023 Dec 23:2023.12.21.572875. Preprint. [Version 1] doi: 10.1101/2023.12.21.572875 (PMC10769365; doi:10.1101/2023.12.21.572875)
Supplement: Supplementary file 1 [file media-1.pdf]

Supplementary file

Structure of the human systemic RNAi defective transmembrane protein 1 (hSIDT1) reveals the conformational flexibility of its lipid binding domain

Vikas Navratna, Arvind Kumar, Jaimin K. Rana, and Shyamal Mosalaganti

Supplementary figures

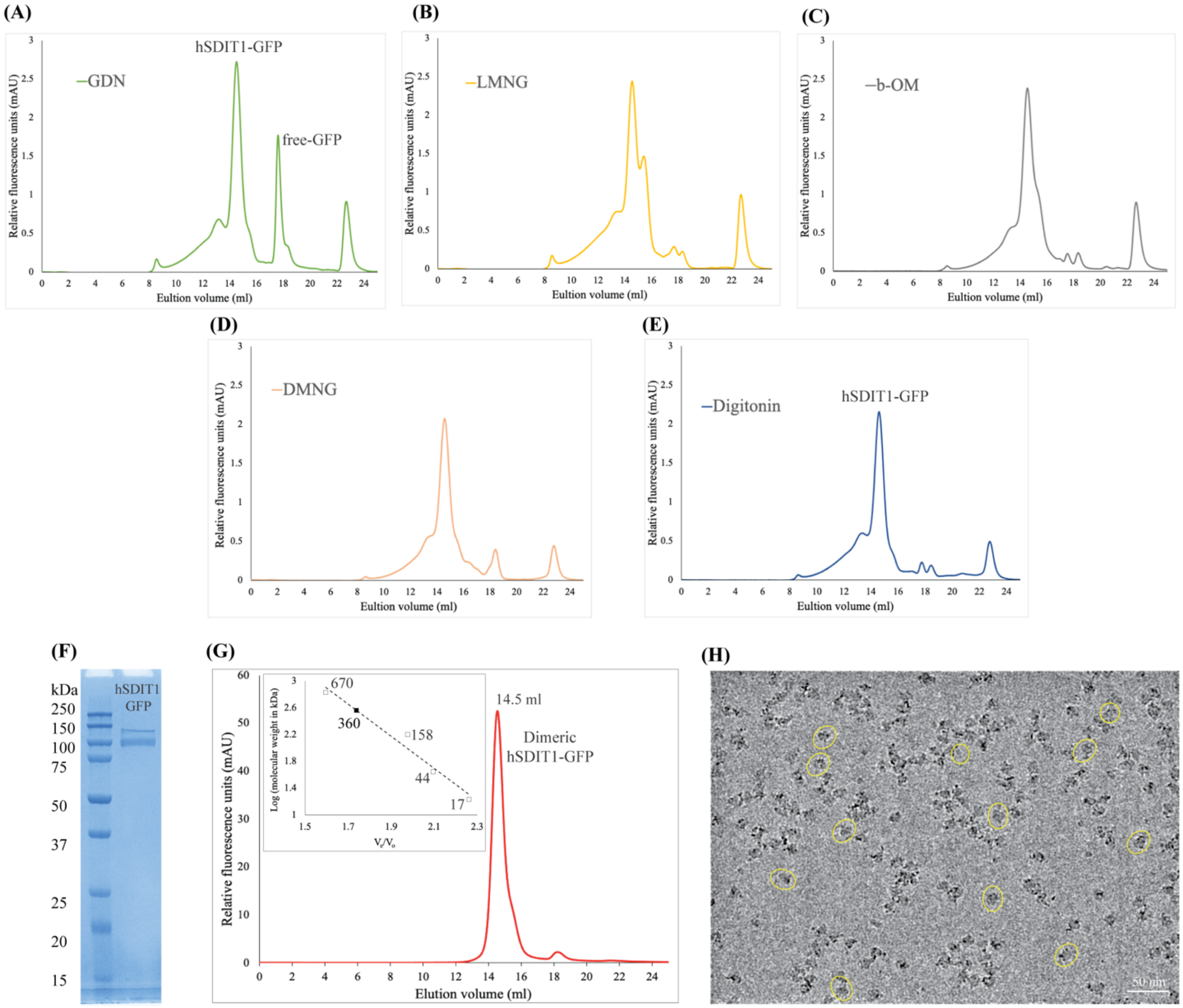

Figure S1: hSIDT1 purification and cryo-EM sample preparation

(A-E) Comparison of the relative solubility and homogeneity in 1% of GDN, LMNG, β-OM, DMNG, and digitonin, respectively. All the detergents were prepared in 25 mM Tris-HCl, pH 7.5, 200 mM NaCl, 1 mM PMSF, 0.8 μM aprotinin, 2 μg/mL leupeptin, and 2 μM pepstatin A. **(F)** SDS-PAGE (12%) showing the purity of hSIDT1-GFP. Although the estimated monomeric

molecular weight of the fusion protein is ~ 124 kDa, like other eukaryotic membrane proteins, hSIDT1 displays anomalous electrophoretic mobility. **(G)** Intrinsic tryptophan fluorescence of purified hSIDT1-GFP was monitored in a size-exclusion chromatography experiment on Superose 6 Increase 10/300 GL column at 0.5 ml/min flowrate. Black square on the standard plot (log of protein molecular weight (kDa) vs. ratio of the elution volume to the void volume (Ve/Vo) indicates that recombinant hSIDT1-GFP fusion, which elutes at 14.5 ml corresponding to a dimer molecular weight of ~360 kDa (hSIDT1-GFP dimer + digitonin micelle). **(H)** Representative micrograph showing vitrified hSIDT1-GFP (yellow circles) at 1 mg/ml on UltrAuFoil holey-gold 300 mesh 1.2/1.3 µm grids, imaged using Titan Krios G4i transmission electron microscope. The protein distribution on the grids, SDS-PAGE, and size-exclusion chromatogram indicated a monodisperse sample preparation.

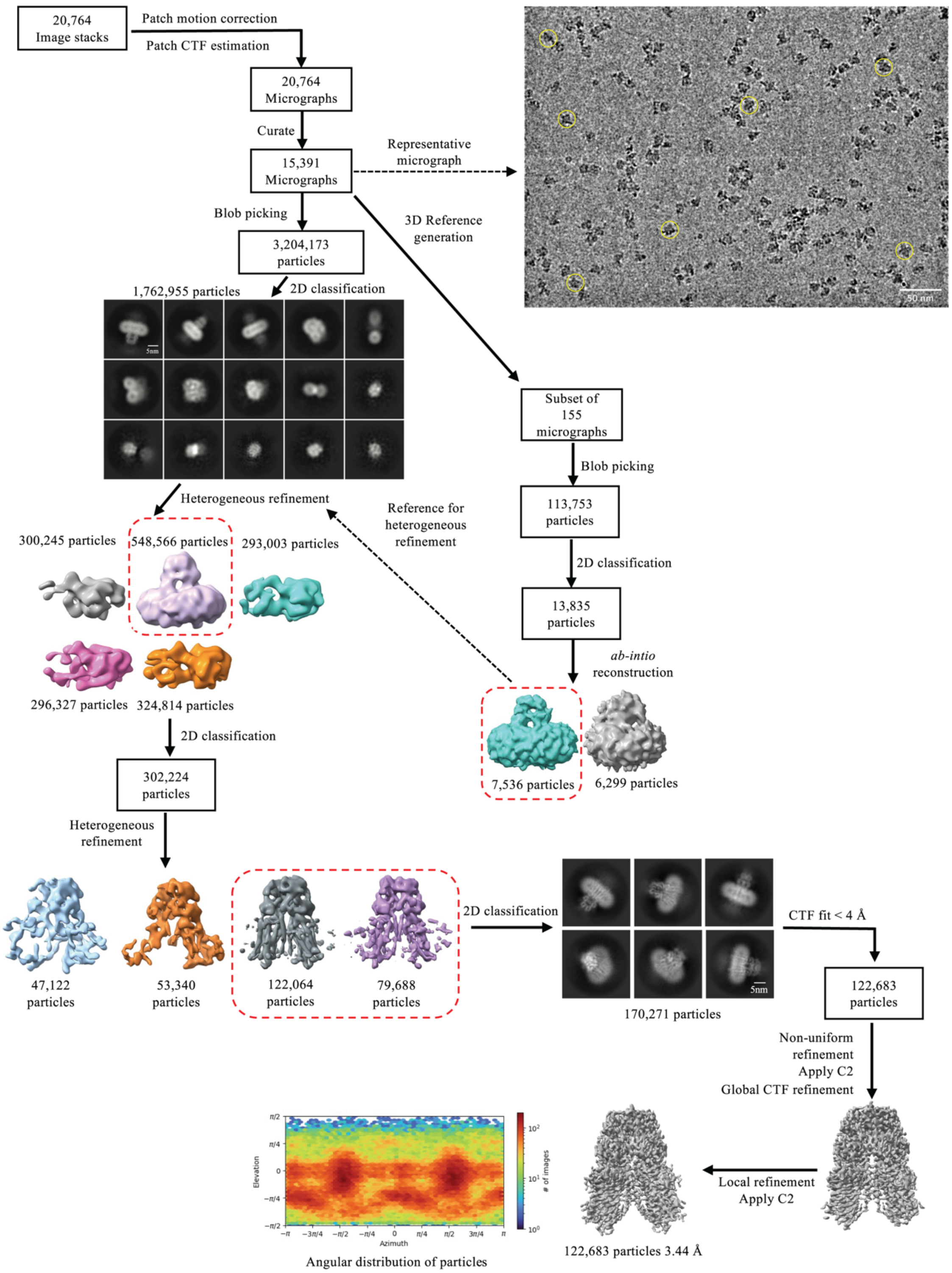

Figure S2: Processing workflow of hSIDT1-GFP cryo-EM data

The data was processed using CryoSPARC (v 4.2.1) [1]. On the top right, a representative motion-corrected micrograph with hSIDT1-GFP particles (yellow circles) is highlighted. A subset of 155

motion-corrected micrographs was selected, and particles were picked by blob picker and cleaned by iterative 2D classification to generate a low-resolution *ab initio* reconstruction to be used as a reference input for heterogeneous refinement of the entire dataset (dashed box and arrow). Briefly, ~3.2 million particles were picked by blob picker, cleaned by 2D classification, and sorted further by heterogeneous refinement. ~548,000 particles from the best class (dashed box) in this heterogeneous refinement were further cleaned up by iterative 2D classification. ~302,000 particles were re-extracted unbinned in a 360 px box and sorted further by iterative heterogeneous refinement and 2D classification jobs. The resultant set of 170,000 clean particles was re-extracted from micrographs with a CTF fit <4 Å to yield a final stack of about 122,000 particles. C2 symmetry was applied at this stage, and non-uniform and CTF refinements were performed to obtain a final map at 3.44 Å [2]. The sharpened and unsharpened maps from this job were used for model building and analyzing the structure of hSIDT1-GFP. The final 3D model was validated using the sharpened map for deposition.

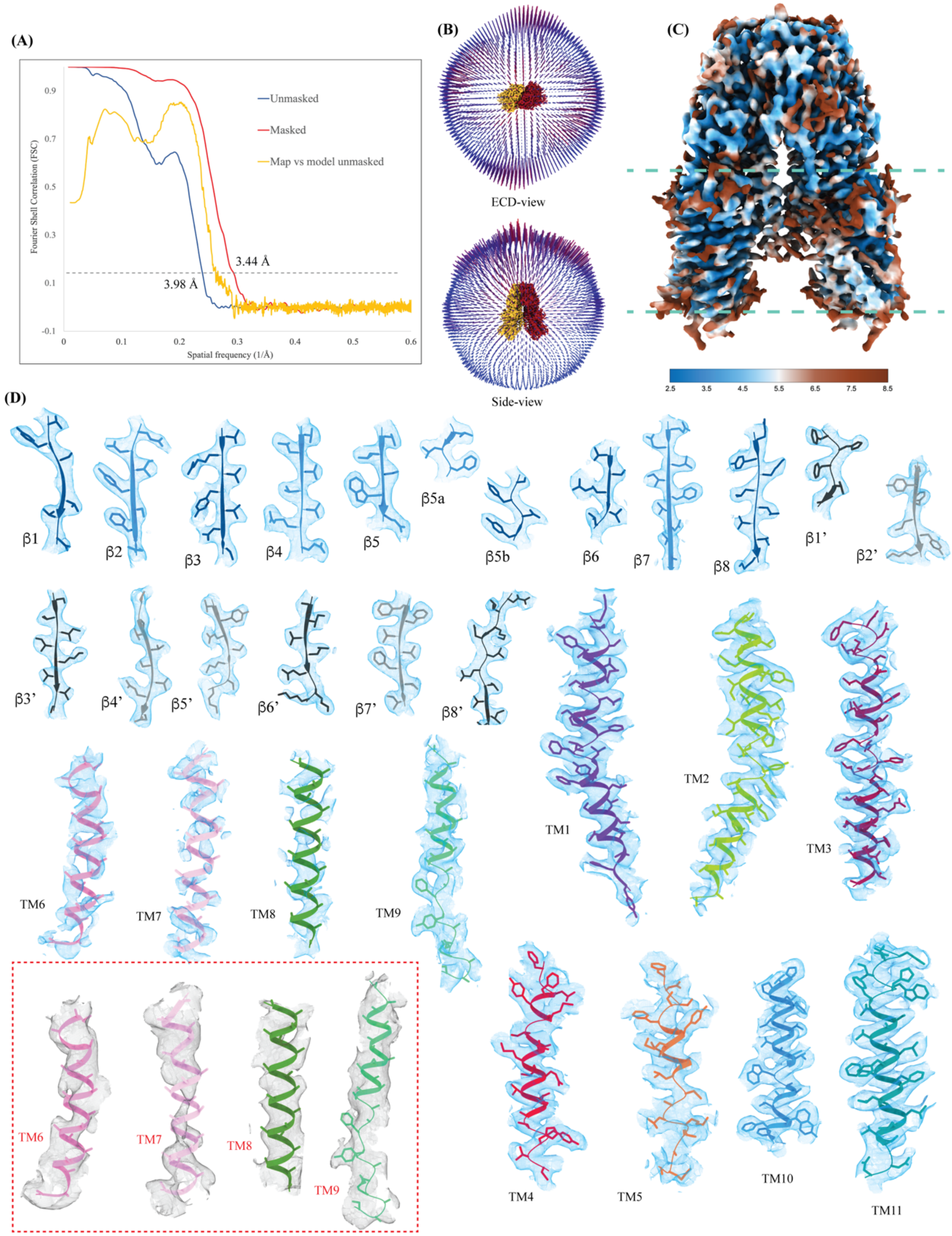

Figure S3: Cryo-EM map quality and model building

(A) The cross-validation Fourier shell correlation (FSC) curves for the final masked hSIDT1-GFP masked (red) and unmasked (blue) refinement maps and the model vs. final unmasked map

(yellow). The gray dashed line indicates the $FSC_{0.143}$ threshold. FSC curves were calculated using Phenix's Mtriage [3]. **(B)** The angular distribution of the particles used in the final reconstruction in ECD- and side-view. **(C)** Estimated local resolution of the final C2 refined map (display level 0.14 in ChimeraX). **(D)** The cryo-EM density of all the secondary structure elements is shown in light blue at a display level 0.25 for the ECD region and 0.1 for the TMD region in ChimeraX [4]. Side chains for almost all the elements could be modeled unambiguously into the density except for TMs 6-8 and the extra-cytosolic half of TM9. In these regions, the Cα traces were placed using an unsharpened map (red dashed box), and the side chains were trimmed to Cb. ECD was modeled into the density using ModelAngelo [5]. The AlphaFold model of hSIDT1 (UniProt ID: Q9NXL6) was used to build the TMD core, and TMs 5-9 were manually built using Coot [6, 7].

(A)

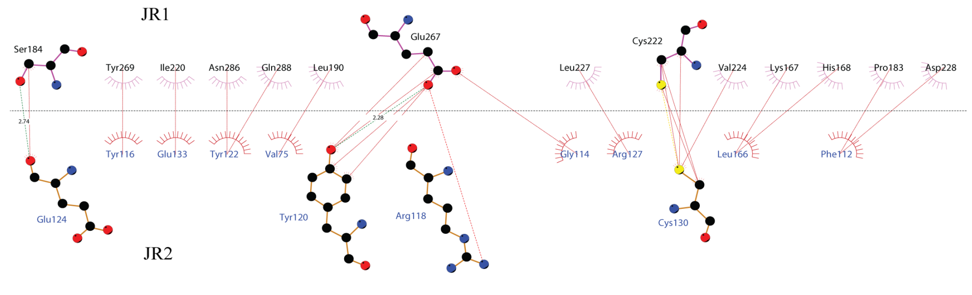

JR1-JR2 interface

(B)

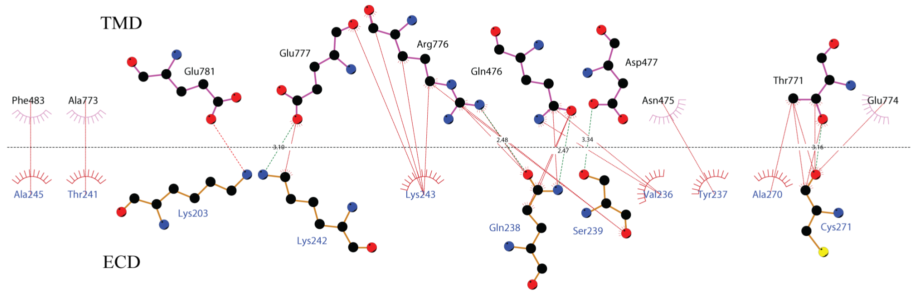

ECD-TMD interface

(C)

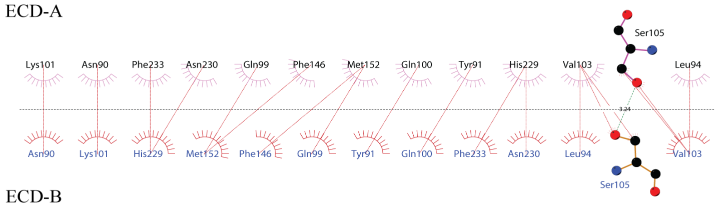

ECD-A-ECD-B interactions at the dimer interface (sub-interface 1 & 2)

(D)

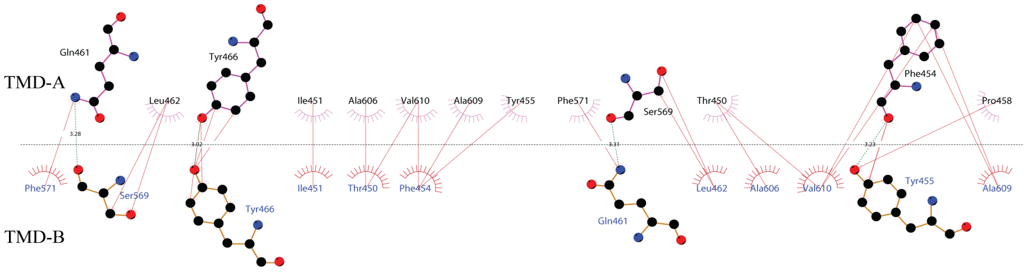

TMD-A-TMD-B interactions at the dimer interface (sub-interface 3)

Figure S4: 2D plots of the critical inter-chain and intra-chain interactions

LigPlot+ was used to generate the 2D depictions of the interactions (<4.5Å) at the **(A)** JR1-JR2 interface, **(B)** ECD-TMD interface, and **(C)** and **(D)** dimer interface [8]**.** Hydrogen bonds are depicted by dashed lines, critical residues are shown as ball & stick models, and amino acids

involved in the non-bonded interactions are indicated as eye lashes. The sulfurs of disulfides are highlighted in yellow.

Supplementary tables

Table S1: Comparison of the sample preparation conditions employed by various studies

	**Qian et al.,** [9]	**Zheng et al.,** [10]	**Hirano et al.,** [11]	**Sun et al.,** [12]	**This study**
	**Expression**				
**Cell type**	HEK293F	Sf9	Expi293F	HEK293F	HEK293sGnTI-
**Expression construct**	Full-length hSIDT2	ΔCL1-hSIDT1 and ΔCL1-hSIDT2	Full-length hSIDT1	Full-length hSIDT1	Full-length hSIDT1-GFP
**Gene transfer**	Transient transfection	Baculoviral transduction	Baculoviral transduction	Transient transfection	Baculoviral transduction
**Cell culture time/temp**	48 h/37 ºC	60 h/27 ºC	96 h/30ºC	12 h/37 ºC<br>48 h/30 ºC	8 h/37 ºC<br>36 h/32 ºC
**Culture volume**	12 L	6 L	-	1L	1 L
	**Purification**				
**Cell lysis buffer**	25 mM HEPES, pH 7.4, 150 mM NaCl, 1.95 µg/ml aprotinin, 1.5 µg/ml pepstatin, and 3 µg/ml leupeptin	50 mM HEPES, pH 7.5, 300 mM NaCl, 1.04 mM AEBSF, 0.8 µM Aprotinin, 50 µM Bestatin, 15 µM E-64, 20 µM Leupeptin, 15 µM Pepstatin A, and 1 mM PMSF	20 mM Tris-HCl, pH 7.0, 150 mM NaCl, and 1X protease inhibitor cocktail	40 mM Tris-HCl, pH 7.5, 150 mM NaCl, 20% glycerol (v/v), and 1X protease inhibitor cocktail	25 mM Tris, pH 7.5, 200 mM NaCl, 0.8 uM aprotinin, 2 ug/ml leupeptin, and 2 uM pepstatin A
**Cell lysis**	-	High-pressure cell disruption	Sonication	High-pressure Homogenizer	Sonication
**Detergent for solubilization prepared in cell lysis buffer**	1% (w/v) DMNG and 0.1% (w/v) CHS	2% (w/v) DDM and 0.2% (w/v) CHS	1.7% (w/v) LMNG or digitonin	1% (w/v) LMNG and 0.1% (w/v) CHS	1% (w/v) Digitonin
**Affinity resin**	Tandem anti-FLAG & Ni-NTA	Anti-FLAG	Anti-FLAG	Anti-FLAG	Strep-Tactin
**Wash buffer**	0.01% GDN in cell lysis buffer	0.02% (w/v) LMNG 0.002% (w/v) CHS in cell lysis buffer, plus 1 mM EDTA	0.01% LMNG or 0.01% GDN 20 mM Tris-HCl, pH 7.0, and 150 mM NaCl	40 mM Tris-HCl, pH 7.5, 150 mM NaCl, 10% glycerol (v/v), 1 mM DTT, 0.5 mM ATP-Mg^{+2}, 0.02% (w/v) GDN	0.5% (w/v) Digitonin, 25 mM Tris, pH 7.5, and 200 mM NaCl
**Elution buffer**	0.01% GDN in cell lysis buffer, plus 300 ug/ml	0.01% (w/v) LMNG	0.01% LMNG or 0.01% GDN 20 mM MES-	40 mM Tris-HCl, pH 7.5, 150 mM NaCl,	5 mM Desthiobiotin in wash buffer

	FLAG peptide (for Anti-FLAG) or 300 mM imidazole for Ni-NTA)	0.001% (w/v) CHS in cell lysis buffer, plus 500 ug/ml FLAG peptide, 50 mM HEPES, pH 7.5, and 300 mM NaCl	NaOH, pH 6.0, 150 mM NaCl, and 5 M LiCl	10% glycerol (v/v), 1 mM DTT, 0.02% (w/v) GDN, plus 200 ug/ml FLAG peptide	
**SEC buffer**	0.006% (w/v) GDN, 25 mM HEPES, pH 7.4, and 150 mM NaCl	0.01% (w/v) LMNG 0.001% (w/v) CHS, 25 mM HEPES, pH 7.5, 150 mM NaCl, and 0.5 mM EDTA	0.01% LMNG or 0.01% GDN 20 mM MES-NaOH, pH 6.0, and 150 mM NaCl	40 mM Tris-HCl, pH 7.5, 150 mM NaCl, and 0.02% GDN	0.5% (w/v) Digitonin, 25 mM Tris, pH 7.5, and 200 mM NaCl
**Protein concentration**	13 mg/ml	3.5 mg/ml	2.5-5.0 mg/ml	17 mg/ml	1 mg/ml
	**Cryo-EM sample**				
**Grid type**	Quantifoil Au 300 mesh, R1.2/1.3	Quantifoil Cu 300 mesh, R0.6/1.0 or Nanodim R1.2/1.3 amorphous nickel titanium alloy (ANTA) grid	Quantifoil Cu 300 mesh, R1.2/1.3	Quantifoil Cu 300 mesh, R1.2/1.3	UltrAufoil Au 300 mesh, R1.2/1.3
**Pretreatments**	Grids glow-discharged	Grids glow-discharged and treated with 0.1% poly L-lysine hydrobromide	Grids glow-discharged	Grids glow-discharged	Grids glow-discharged, and 100 uM FOM added to protein sample
**Blotting**	3 s, 8 ºC, 100% humidity	2.5-3 s, 8 ºC, 100% humidity	4 s, 6 ºC, 100% humidity	4s, 6 ºC, 100% humidity	2.5 s, 18 ºC, 100% humidity
	**Cryo-EM data collection**				
**Microscope**	Titan Krios (K3, 300 kv)	Titan Krios (K2/K3, 300 kv)	Titan Krios (K3, 300 kv), CDS mode	Titan Krios (K3, 300 kv)	Titan Krios (K3, 300 kv)
**Pixel size**	1.08 Å/px	0.82 Å/px	0.83 Å/px	0.535 Å/px	0.85 Å/px
**Total dose**	50 $e^-/Å^2$	60 $e^-/Å^2$	66 $e^-/Å^2$	50-60 $e^-/Å^2$	50 $e^-/Å^2$
**Defocus range**	-1.3 to -1.8 um	-1.2 to -1.8 um	-	-1.5 to -2.0 um	-1.0 to -2.5 um

Table S2: Comparison of the Cα RMSDs of hSIDT1-GFP (8V38) superposition on the existing ChUP family structures

	8JUL	8JUN	8HIIP	8HKE	7Y63	7Y68	7Y69
**ECD dimer Cα RMSD**	1.2 Å	1.2 Å	6.7 Å	7.0 Å	2.2 Å	1.5 Å	1.4 Å
**TMD dimer Cα RMSD**	4.2 Å	2.9 Å	6.0 Å	5.7 Å	3.6 Å	3.4 Å	3.5 Å
**Full dimer Cα RMSD**	4.1 Å	3.1 Å	8.2 Å	9.0 Å	3.3 Å	3.3 Å	3.3 Å
**ECD chain A Cα RMSD**	1.2 Å	1.2 Å	6.7 Å	9.9 Å	2.2 Å	1.5 Å	1.5 Å
**TMD chain A Cα RMSD**	4.2 Å	2.9 Å	6.0 Å	5.7 Å	3.6 Å	3.4 Å	3.4 Å
**Full chain A Cα RMSD**	4.1 Å	3.2 Å	8.2 Å	9.0 Å	3.3 Å	3.3 Å	3.3 Å

Supplementary references:

1. Punjani A, Rubinstein JL, Fleet DJ, Brubaker MA. cryoSPARC: algorithms for rapid unsupervised cryo-EM structure determination. Nat Methods. 2017;14(3):290-6. Epub 20170206. doi: 10.1038/nmeth.4169. PubMed PMID: 28165473.

2. Punjani A, Zhang H, Fleet DJ. Non-uniform refinement: adaptive regularization improves single-particle cryo-EM reconstruction. Nat Methods. 2020;17(12):1214-21. Epub 20201130. doi: 10.1038/s41592-020-00990-8. PubMed PMID: 33257830.

3. Afonine PV, Klaholz BP, Moriarty NW, Poon BK, Sobolev OV, Terwilliger TC, et al. New tools for the analysis and validation of cryo-EM maps and atomic models. Acta Crystallogr D Struct Biol. 2018;74(Pt 9):814-40. Epub 20180903. doi: 10.1107/S2059798318009324. PubMed PMID: 30198894; PubMed Central PMCID: PMCPMC6130467.

4. Meng EC, Goddard TD, Pettersen EF, Couch GS, Pearson ZJ, Morris JH, et al. UCSF ChimeraX: Tools for Structure Building and Analysis. Protein Sci. 2023:e4792. Epub 20230929. doi: 10.1002/pro.4792. PubMed PMID: 37774136.

5. Jamali K, Kall L, Zhang R, Brown A, Kimanius D, Scheres SHW. Automated model building and protein identification in cryo-EM maps. bioRxiv. 2023. Epub 20230516. doi: 10.1101/2023.05.16.541002. PubMed PMID: 37292681; PubMed Central PMCID: PMCPMC10245678.

6. Emsley P, Lohkamp B, Scott WG, Cowtan K. Features and development of Coot. Acta Crystallogr D Biol Crystallogr. 2010;66(Pt 4):486-501. Epub 20100324. doi: 10.1107/S0907444910007493. PubMed PMID: 20383002; PubMed Central PMCID: PMCPMC2852313.

7. Varadi M, Anyango S, Deshpande M, Nair S, Natassia C, Yordanova G, et al. AlphaFold Protein Structure Database: massively expanding the structural coverage of protein-sequence space with high-accuracy models. Nucleic Acids Res. 2022;50(D1):D439-D44. doi: 10.1093/nar/gkab1061. PubMed PMID: 34791371; PubMed Central PMCID: PMCPMC8728224.

8. Laskowski RA, Swindells MB. LigPlot+: multiple ligand-protein interaction diagrams for drug discovery. J Chem Inf Model. 2011;51(10):2778-86. Epub 20111005. doi: 10.1021/ci200227u. PubMed PMID: 21919503.

9. Qian D, Cong Y, Wang R, Chen Q, Yan C, Gong D. Structural insight into the human SID1 transmembrane family member 2 reveals its lipid hydrolytic activity. Nat Commun. 2023;14(1):3568. Epub 20230615. doi: 10.1038/s41467-023-39335-2. PubMed PMID: 37322007; PubMed Central PMCID: PMCPMC10272179.

10. Zheng L, Yang T, Guo H, Qi C, Lu Y, Xiao H, et al. Cryo-EM structures of human SID-1 transmembrane family proteins and implications for their low-pH-dependent RNA transport

activity. Cell Res. 2023. Epub 20231106. doi: 10.1038/s41422-023-00893-1. PubMed PMID: 37932445.

11. Yoshinori Hirano UO, Ryota Sato, Kensuke Miyake, Toshiyuki Shimizu, . Cryo-EM analysis reveals human SID1 transmembrane family member 1 dynamics underlying lipid hydrolytic activity. Research Square https://doi.org/10.21203/rs.3.rs-3267442/v12023.

12. Sun CR, Xu D, Yang F, Hou Z, Luo Y, Zhang CY, et al. Human SIDT1 mediates dsRNA uptake via its phospholipase activity. Cell Res. 2023. Epub 20231106. doi: 10.1038/s41422-023-00889-x. PubMed PMID: 37932444.
